# Circulation of Japanese Encephalitis Virus in Pigs and Mosquito Vectors within Can Tho City, Vietnam

**DOI:** 10.1371/journal.pntd.0002153

**Published:** 2013-04-04

**Authors:** Johanna F. Lindahl, Karl Ståhl, Jan Chirico, Sofia Boqvist, Ho Thi Viet Thu, Ulf Magnusson

**Affiliations:** 1 Department of Clinical Sciences, Division of Reproduction, Swedish University of Agricultural Sciences, Uppsala, Sweden; 2 Department of Biomedicine and Veterinary Public Health, Swedish University of Agricultural Sciences, Uppsala, Sweden; 3 Department of Virology, Immunology and Parasitology, National Veterinary Institute, National Veterinary Institute, SVA, Uppsala, Sweden; 4 Department of Veterinary Medicine, Can Tho University, Can Tho City, Vietnam; United States Army Medical Research Institute of Infectious Diseases, United States of America

## Abstract

Japanese encephalitis virus (JEV) is a mosquito-borne, zoonotic flavivirus causing encephalitis in humans and reproductive disorder in pigs. JEV is present in large parts of Asia, where urbanization is high. Households within and outside Can Tho city, South Vietnam, were selected to monitor circulation of JEV. A nested RT-PCR was established to detect the presence of JEV in mosquitoes whereas sera from pigs belonging to households within the province were analyzed for the presence of antibodies to JEV. A total of 7885 mosquitoes were collected and divided into 352 pools whereof seven were JEV-positive, six of which were collected within the city. Fragments from four pools clustered with JEV genotype III and three with genotype I. Of the 43 pigs sampled inside the city 100% had JEV antibodies. Our study demonstrates exposure to JEV in pigs, and co-circulation of JEV genotype I and III in mosquitoes within an urban environment in South Vietnam. Thus, although JEV has mainly been considered a rural disease, the potential for transmission in urban areas cannot be ignored.

## Author Summary

Japanese encephalitis (JE) is a serious disease, especially in children, in large parts of Asia. It is transmitted by mosquitoes and mainly known as a disease in the rural, rice-producing areas in South and Southeast Asia. Here, the authors show that of 43 pigs sampled in Can Tho city, South Vietnam, all had antibodies to JE virus (JEV), and since some of these were born in the city, they must have been infected within the urban area. Mosquitoes were collected during night at households both in the city and in a rural area, using traps that attract mosquitoes with light. The mosquitoes were analyzed to see if they had JEV in them. Two different types of JEV were found in mosquito samples collected at urban households. One of these, genotype I, has been emerging in Northern Vietnam and was now shown to be circulating together with the older genotype III. These findings suggest a risk of JEV transmission from pigs to humans in urban areas, or in other words, that JE should not be regarded as a rural disease only.

## Introduction

Japanese encephalitis (JE) is a zoonotic disease spread over large parts of Asia. It is one of the most important arboviral encephalitis in humans, with an estimated 10 million cases over the last 60 years, with 30% case fatality [1]. Pigs and wading birds are amplifying hosts of the causative Japanese encephalitis virus (JEV), and do not display clinical signs, except for pregnant sows that may abort or have stillborn piglets [2], [3]. Japanese encephalitis virus is a mosquito-borne flavivirus which is divided into five genotypes [4], and the virus has been isolated from more than 25 mosquito species, although not all are equally important in the epidemiology of JEV [5]. One of the most important vectors is *Culex tritaeniorhynchus*, a zoophilic mosquito that commonly breeds in irrigated rice fields, and therefore the disease is mainly considered rural [1], [3], [6].

Keiser et al. [6] calculated that 1.9 billion people live in rural areas with endemic or epidemic JE. Today, however, more than half of the world's population live in cities [7], [8] and the urbanization, especially in low-income countries, creates needs and possibilities for urban animal keeping to supply city inhabitants with food. Therefore, transmission of emerging zoonotic diseases in urban areas is increasingly important. Since some of the most populated cities in the world are in JE infested countries, the number of people at risk would increase dramatically if JEV is also transmitted in urban areas. It has been shown that two prerequisites for spreading JEV, the presence of competent vectors and the main amplifying host (the pig), are met in urban settings [9], [10]. In an urban area, the vector *Cx. tritaeniorhynchus* has been shown to increase in number by the presence of pigs, whereas the number of another vector, *Culex quinquefasciatus*, increases by the presence of humans [10]. However, the presence of JEV in urban areas has not been studied extensively previously, although previous studies in other cities in Asia have shown seropositivity in humans [11], [12].

In Vietnam, the land area used for rice production is increasing along with pig production, two factors likely to contribute to increased transmission of JEV [13]. Cases of encephalitis in humans are usually reported as acute encephalitis syndrome in Vietnam, and the incidence in south Vietnam has been 1.9 cases annually per 100 000 inhabitants between 1998 and 2007, with a mean case fatality of 6.4% [14]. One of the regions with the most JE cases, the Mekong Delta region [9], [15], has both extensive pig farming and rice production and disease is present all year [16], [17]. Although JEV is known to circulate in the rice-producing rural areas here, little is known about the circulation of JEV within urban areas.

The aim of the present study was to investigate the presence of JEV in pigs and vectors in a city in an endemic area, in order to contribute to the risk assessment of JE in humans. Pigs within and outside Can Tho city in the Mekong delta region, Vietnam, were examined serologically for JEV, and mosquitoes from the same locations were investigated for presence of JEV viral RNA. A high seroprevalence in pigs kept in the urban area is reported here, as well as the presence of JEV genotype I and III in mosquitoes collected in the city.

## Materials and Methods

### Ethical statement

All animals in this study were treated according to the ethical standards of Can Tho University, Vietnam, and all animal handling was approved by the head of the Department for Veterinary Medicine, Can Tho University. Blood collections were performed by jugular venopuncture, which is the international recommended method [18] and also adhere to the Swedish guidelines for sampling blood from pigs in research [19]. A vacutainer system collecting maximally 10 ml was used. Household and pig owners were informed about the purpose and the methods of the study, and provided oral informed consent and answered questionnaires as a written consent.

### Location

Can Tho city is a central province in the Mekong delta region, comprised of eight districts, which are subdivided into wards. The most urbanized district is Ninh Kieu; it is comprised of the actual Can Tho city, which itself is subdivided in 13 wards, and has a total human population density of 7500 persons/km^2^ and a pig density of 94 pigs/km^2^ [20], and the more rural districts of the province have human population densities between 380 and 1400 persons/km^2^ and pig densities between 65 and 123 pigs/km^2^.

Ten urban wards in Can Tho city with different ratio of pigs/people were selected to represent different parts of the city. In these wards, households were included if sampling was allowed and if it was possible to affix traps for mosquito collections. In total 14 urban households that kept pigs and five households without pigs were included. Three households at pig farms in the rural Co Do district were also included as a comparison.

### Mosquito collections and RNA extraction

Mosquitoes were collected during two three-week periods in February–March (spring) and October–November 2009 (fall), using un-baited CDC mini light traps (Bioquip Products, California, USA) as described by Lindahl et al. [10]. Briefly, two traps were operated from dusk to dawn in the same household, close to human dwellings, and if the household had pigs, one of these traps was placed close to the pigs, immediately adjacent to the pig pen. Mosquitoes were identified according to Reuben et al. [21]. In catches containing more than 300 mosquitoes, 300 were identified to species, since this number always represented more than 10% of the catch and thus considered sufficient to estimate the existing species composition. The remaining non-identified specimens were counted and pooled unsorted.

Mosquitoes were pooled according to collection site with 1–60 mosquitoes per pool. To each pool, TRIzol Reagent (Invitrogen, Carlsbad, CA), corresponding to at least 10 times the total mosquito volume, was added. The mosquito pools were then stored at −20°C before analysis, except during transport to the National Veterinary Institute, Uppsala, Sweden, when samples were kept cold in a box with ice packs.

The mosquito pools were subsequently homogenized in a TissueLyzer (Qiagen GmbH, Hilden, Germany) for 2×1 min at 30 rpm after addition of one 5 mm steel ball to each tube. Extraction of the homogenates was performed according to the manufacturer's protocol for TRIzol Reagent with dilution of the resulting pellet in 20 µl of nuclease-free water and subsequent storage at −70°C.

### Establishment of a nested RT-PCR protocol

To create a sensitive method for JEV detection in mosquito samples a nested RT-PCR protocol was established. When mosquito pools are analyzed for the presence of viral RNA using PCR, there is a risk for false negative results due to inhibition caused by the mosquitoes [22], [23]. To assess the inhibitory effect of mosquitoes, Swedish mosquitoes, assumed to be negative to flaviviruses as no known mosquito-borne flaviviruses are transmitted in Sweden [24], were used for spiking. Mosquitoes were caught by hand-net in Uppsala. 750 µl of TRIzol Reagent (Invitrogen, California, USA) were added to pools with 5 and 50 mosquitoes and homogenized as above. The Nakayama JEV strain, provided as a TRIzol Reagent suspension by the Swedish Institute for Control of Communicable Diseases (Solna, Sweden), was used. The suspension was diluted 1∶100 and 1∶1000 in TRIzol Reagent, and 1∶1000 in the homogenates of 5 and 50 mosquitoes, in order to mimic pools with low viral contents under field conditions.

RNA was extracted from all spiked virus suspensions, using TRIzol Reagent as described for the mosquito samples. All extractions were thereafter diluted in RNA safe buffer (RSB) [25] in dilution series to evaluate sample dilution as a method to avoid inhibition in a simple and cost-effective way. To further evaluate if inhibition occurs already in the extraction step, the extraction from the 1∶100 dilution of virus in TRIzol Reagent was diluted 1∶10 in the extraction from 50 homogenized mosquitoes without JEV, to reach the same concentration of extracted viral RNA as the other extractions.

The nested RT-PCR was performed using Agpath-id One step RT-PCR and Path-id PCR (Applied Biosystems, Foster City, CA, USA) according to the manufacturer's instructions with T3000 Thermocycler (Biometra, Goettingen, Germany) and Rotorgene3000 (Qiagen/Corbett Research, Sydney, Australia) respectively. The outer set of primers, *emf1* and *vd8* (Table 1), amplify an approximately 650 bp sequence from the non-structural protein 5 (NS5)-3′untranslated region (UTR) of all flaviviruses [26], whereas the inner set of primers and probe was specific for the NS5 region of JEV [27]. The probe (Table 1) was modified with a degeneration in the middle to improve sensitivity, since preliminary studies indicated a variation in nucleotide sequence (results not shown).

**Table 1:** Primers and probe in a nested RT-PCR for Japanese encephalitis virus (JEV).

Primers used	Sequence 5′- 3′	Position in JEV genome	Reference
Outer forward (emf1)	TGGATGACSACKGARGAYATG	10099–10119	Pierre et. al (1994)
Outer reverse (vd8)	GGGTCTCCTCTAACCTCTAG	10771–10752	Pierre et. al (1994)
Inner forward	ATCTGGTGYGGYAGTCTCA	10224–10242	Pyke et al. (2004)
Inner reverse	CGCGTAGATGTTCTCAGCCC	10286–10267	Pyke et al. (2004)
Probe	FAM-CGGAACGCGAWCCAGGGCAA-TAMRA	10244–10263	Pyke et al. (2004)

The first RT-PCR was set up with final primer concentrations of 160 nM, in a 25 µl reaction with 2 µl RNA template. The subsequent qPCR was performed with 1 µl of the product from the first PCR reaction in a 25 µl reaction using a primer concentration of 400 nM and the probe in a concentration of 150 nM. All Vietnamese mosquito samples were diluted with RSB and run in two dilutions (1∶10 and 1∶100), to avoid inhibition, according to the results using the spiked mosquitoes (Table 2).

**Table 2:** Nested RT-PCR results of samples spiked with Japanese encephalitis virus.

Sample	Dilution	Nested RT-PCR	
		Positive/runs in total	Mean ct*
JEV diluted 1∶1000 in TRIzol	1∶1	2/2	14.7
Reagent	1∶10	2/2	16.6
	1∶100	3/3	17.2
	1∶1 000	3/3	21.2
	1∶10 000	2/2	26.3
	1∶100 000	2/2	28.6
JEV diluted 1∶1000 in	1∶1	0/3	
homogenate of 5 mosquitoes	1∶10	2/3	34.0
in TRIzol Reagent	1∶100	3/3	22.0
	1∶1 000	2/3	23.2
	1∶10 000	3/3	31.2
	1∶100 000	1/3	34.2
JEV diluted 1∶1000 in	1∶1	0/2	
homogenate of 50	1∶10	0/2	
mosquitoes in TRIzol	1∶100	2/2	20.6
Reagent	1∶1 000	2/2	24.6
	1∶10 000	2/2	29.6
	1∶100 000	2/2	36.4

* Mean cycle threshold for the positive runs.

### Sequencing and phylogenetic analysis

The product from the first RT-PCR was reamplified using the outer forward primer (*emf1*) and the inner reverse primer. The product was visualized on a 1.5% agarose gel, excised and extracted using QIAquick Gel extraction kit (Qiagen Gmbh, Hilden, Germany) according to the manufacturer's protocol. Sequencing was performed using ABI PRISM Big Dye Terminator Cycle Sequencing v3.1 Ready Reaction kit (Perkin Elmer, Waltham, MA, USA) on an ABI PRISM 310 genetic analyzer (Applied Biosystems, Foster City, California, USA) according to the manufacturer's instructions. The resulting 133 bp sequences were aligned using BioEdit Sequence Alignment Editor [28] with reference sequences from different JEV genotypes derived from the NCBI GenBank database. Phylogenetic analysis was performed in MEGA version 5 [29] using the neighbor-joining method. Bootstrap probabilities were calculated using 10 000 replicates and evolutionary distances were computed using the p-distance method. To verify that the reference strains clustered the same way, a phylogenetic tree was also created the same way with the reference strains of their entire length.

### Infection rates in mosquitoes

The infection rate in the mosquitoes was calculated using two methods. The minimum infection rate (MIR) in the mosquitoes was calculated as the number of positive pools divided with the total number of mosquitoes, assuming that at least one mosquito was positive in the positive pool. The maximum likelihood estimate (MLE) for the mosquito infection rate was calculated using the software by Biggerstaff [30] (www.cdc.gov/ncidod/dvbid/westnile/software.htm). As both blood-fed and unfed mosquitoes were used in the analysis, the MLE here may be an overestimation of the infection rate, since some of the mosquitoes may have had virus only in a blood meal.

### Serological analyses

Blood sampling of pigs was performed during the sampling period in the rainy season, and restricted to sows and gilts over six months of age, hereafter referred to as female pigs. As many female pigs as the owner would allow, up to eleven pigs per household, were sampled. Blood samples were collected from the jugular vein using vacutainers, and kept cool with ice packs, until centrifuged the same day, after which the serum was stored at −20°C. Samples were transported frozen in a box with ice packs and inactivated for 60 min at 60°C at arrival, and stored at −20°C until analyses.

**Figure 1:**
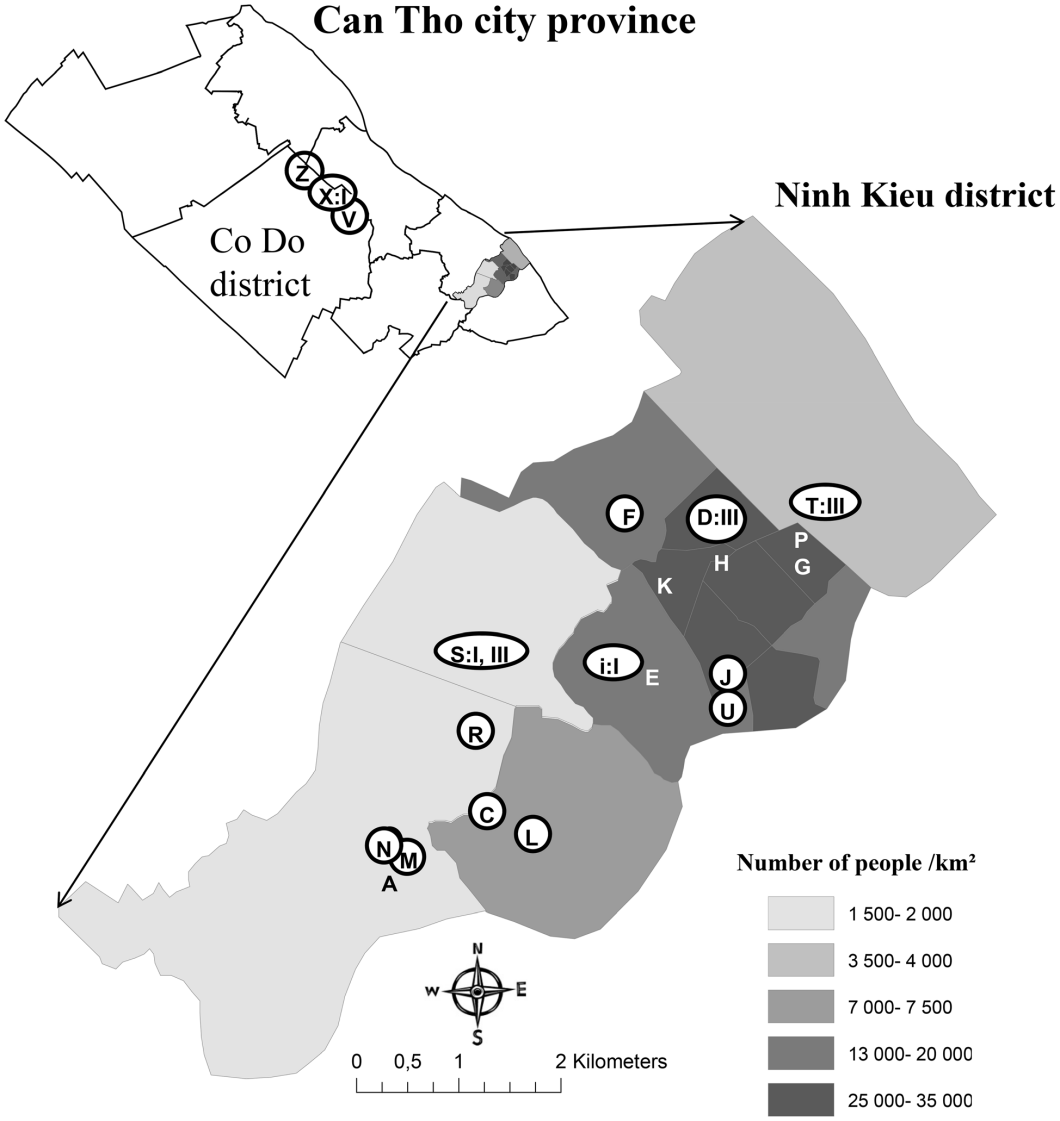
Map showing human population densities in the urban Ninh Kieu district, Can Tho city, Vietnam. Households included for mosquito collections are identified with letters and circles show households where blood samples were also collected from pigs. In households where JEV was detected within the city, this is shown by the roman number for the JEV genotype. The insert of the entire Can Tho city province show collection sites in Co Do district (according to district boundaries in 2009).

At the time for blood sampling, pig keepers were interviewed by a native Vietnamese using a written questionnaire. For every pig, data was collected on age, breed, parity, how many matings that were required to achieve the last pregnancy, the number of piglets born in total and alive in the last litter, if the sow had ever aborted or had stillborn piglets, if she ever had weak born or piglets displaying neurological symptoms at birth (e.g. shivering), how long she had been in the household and vaccination routines. The locations of the households where pigs were sampled are shown in Figure 1.

Serological analyses were performed using competitive IgG ELISA and IgM MAC ELISA as previously described [31], [32] and antigen, antibodies, conjugate and controls were provided from Australian Animal Health Laboratories (AAHL, Geelong, Victoria, Australia). All samples were tested twice on plates coated on different days. In the IgG ELISA a sample was considered positive if the inhibition was above 65% on both tests compared to the negative control. If a sample yielded different results it was considered inconclusive. In the IgM MAC ELISA, the ratio of sample optical density (OD) to the OD-value of the lowest concentration of the positive control was calculated. A sample was considered positive if it had a ratio greater than one.

Swedish pig sera were used to verify the specificity of the ELISAs. The sera originated from routine surveillance of infectious diseases in Swedish control programs. To evaluate the effect of heat inactivation on the specificity of the assays, the Swedish samples were aliquoted and one part was inactivated for 56°C for 60 min, and changes in OD compared to the unheated part, run on the same plate, were monitored.

## Results

### Mosquito inhibition of RT-PCR

The results of the dilution series with Swedish mosquitoes spiked with JEV are shown in Table 2. It was not possible to detect JEV in spiked Swedish mosquito pools unless they were diluted. In a pool with five mosquitoes it was necessary to dilute the extracted RNA 1∶10, and in pools with 50 mosquitoes 1∶100. The same results were obtained if extracted JEV RNA was added after the RNA extraction of the mosquitoes (results not shown).

### Japanese encephalitis virus RNA in mosquitoes from Can Tho city

A total of 7885 mosquitoes, divided into 352 pools, were screened for JEV. Six mosquito pools from four of the urban households were positive for JEV by the nested PCR (Table 3). One positive mosquito pool was found in one of the rural households.

**Table 3:** Households in Can Tho city with mosquito pools positive for Japanese encephalitis virus.

			Population/km^2^				
Sample	Period	Location	Humans	Pigs	Pigs in household	No. of mosquitoes	Genotype
Household D	Spring	Ninh Kieu	31 447	26	11	9 *Cx. tritaeniorhynchus*	III
Household D	Fall	Ninh Kieu	31 447	26	14	39 *Cx. tritaeniorhynchus*	III
Household T	Fall	Ninh Kieu	3 706	30	4	30 *Cx. quinquefasciatus*	III
Household I	Spring	Ninh Kieu	12 333	17	26	50 unidentified	I
Household S	Fall	Ninh Kieu	1 703	205	110	50 unidentified	III
Household S	Fall	Ninh Kieu	1 703	205	110	50 unidentified	I
Household X	Fall	Co Do	500	20	134	25 *Cx. tritaeniorhynchus*	I

Minimum infection rate was 1.0 per 1000 mosquitoes (excluding identified males, 95% CI 0.25–1.71). The MLE for infection rate was 1.0 per 1000 mosquitoes. The MLE in *Cx. tritaeniorhynchus* was 1.6 per 1000 mosquitoes and in *Cx. quinquefasciatus* 1.3 per 1000 mosquitoes. When MLE was calculated separately for mosquitoes collected within the city, it was lower, except for *Cx. quinquefasciatus* (Table 4).

**Table 4:** Maximum likelihood estimate (MLE) for Japanese encephalitis virus in Can Tho city.

	MLE per 1000 mosquitoes	95% Confidence interval	MLE urban* per 1000 mosquitoes	95% Confidence interval
All species	1.0	0.4–2.0	0.9	0.4–1.8
(including unidentified mosquitoes)				
*Cx. tritaeniorhynchus*	16	0.4–4.4	1.2	0.2–3.8
*Cx. quinquefasciatus*	1.3	0.1–6.3	1.4	0.1–6.6

* only including Ninh Kieu district, the urban area of Can Tho city.

All positive mosquito pools contained both blood-filled mosquitoes and mosquitoes without visible blood content, and all pools had been collected close to the pigs. At two households, I and S (Table 3), the positive mosquito pools contained non-identified specimens. The 300 identified mosquitoes from the corresponding collection at household I consisted of 46% *Cx. tritaeniorhynchus* and 36% *Cx. gelidus*. The 300 identified mosquitoes at household S consisted of 78% *Cx. tritaeniorhynchus*.

**Figure 2:**
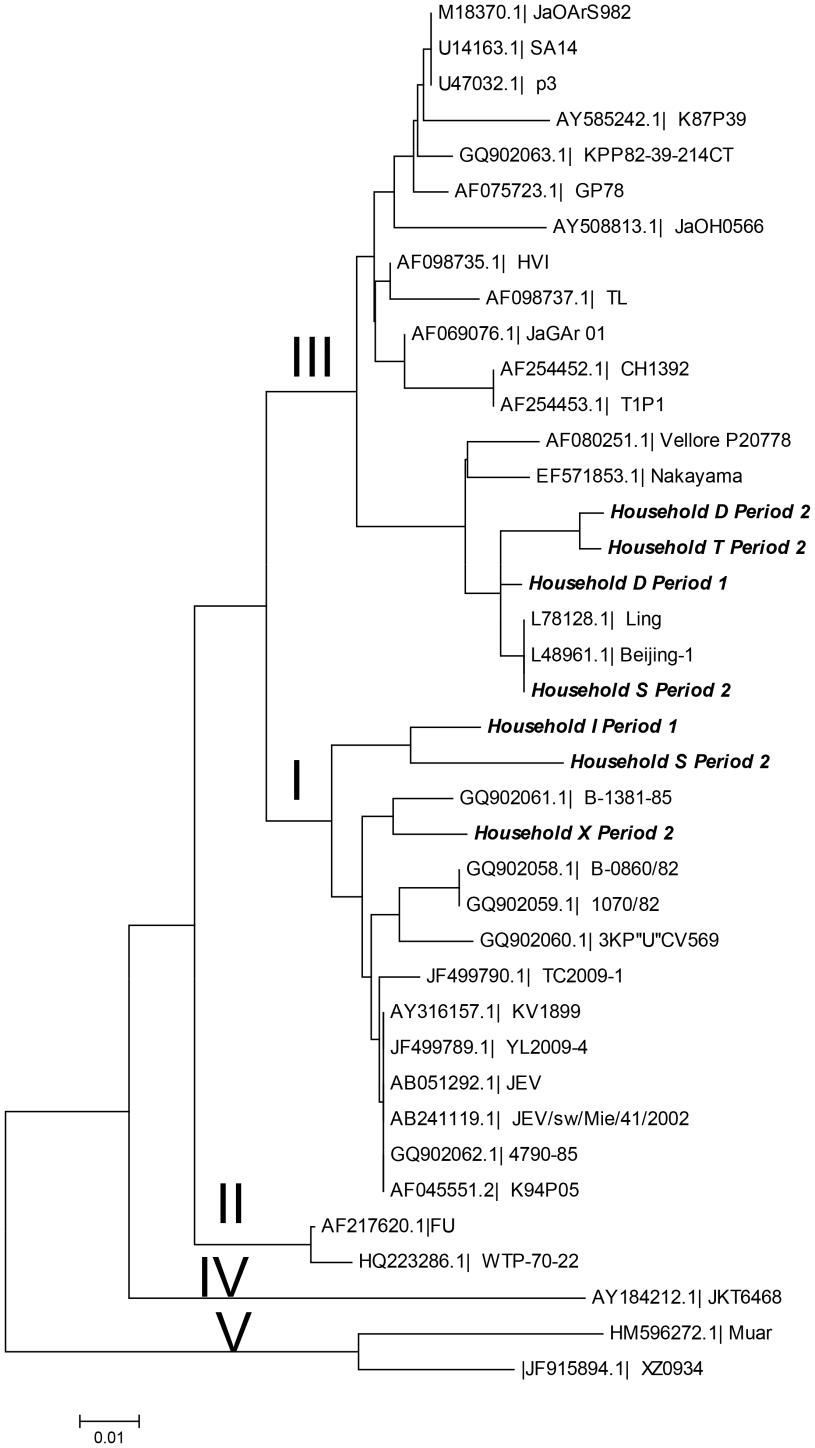
Phylogenetic tree showing the 5 genotypes of Japanese encephalitis virus (I-V). The seven positive mosquito pools (in italics) are named after the household.

The phylogenetic analysis showed that three sequences clustered with genotype I strains, and four with genotype III strains (Figure 2). The pairwise sequence comparison between the fragments showed between 90 and 99% similarity. At household S, sequence fragments from the two positive samples clustered with different genotypes in the phylogenetic tree. The location of samples with the different genotypes within Ninh Kieu district is shown in Figure 1.

### Serology

All 43 female pigs from the urban households and 30 out of the 31 from rural households were positive in the IgG ELISA giving an overall seroprevalence of 99% (95% CI 96%–100%). The test result from one sample was considered inconclusive. All samples tested negative or inconclusive in the IgM ELISA. According to the pig keepers, 24 of the 43 pigs in the urban area and 28 of the 31 pigs in the rural area originated from the household.

### Reproductive disorders in female pigs

In the sows that had at least had one litter (n = 51), repeated estrus was reported for 29%, 8% had aborted, 29% had stillborn or mummified fetuses and 24% weak born piglets, while 4% delivered piglets that shivered at birth. Out of the 51 sows, 28 had never shown any of these symptoms.

## Discussion

Here we demonstrate the concurrent circulation of two JEV genotypes in mosquitoes within the urban area of Can Tho city and extensive seropositivity in pigs born in the city. Worldwide, demographic changes, human behavior, and increased globalization are suggested drivers for emergence of arboviruses such as JEV [33]. However, increased urbanization and the establishment of anthrophilic vectors have been suggested to be the most important catalysts [34]. Even though human JE cases have been shown to occur in cities without extensive pig keeping [11], the increases in the number of vectors close to pigs in urban areas [10], and the fact that all positive mosquito pools had been collected close to pigs in the present study, together indicate increased risks associated with pigs in urban animal farming.

In the present study JEV-RNA fragments from positive mosquito pools clustered with isolates of both genotypes I and III. Genotyping is normally based on sequencing of the E or the prM genes, which both have extensive variation, whereas NS5, the virus polymerase, is highly conserved between viral strains [35], which is confirmed by the limited variation between the sequences found here. Two of the positive samples from urban households were highly similar to the one at the rural household X, 20 km away. Although JEV likely is active throughout the area, explanations for this similarity could be a trade of viremic pigs, or that infected vectors or birds move between rural and urban areas.

Genotype III was previously the most common genotype in Vietnam, but, through sequencing of old and new isolates from northern Vietnam, it has been shown that genotype III gradually has been superseded by genotype I [36], a shift that has been observed in other Asian countries as well [37], [38]. The results of the present study indicate that there is a co-circulation of the two genotypes in southern Vietnam.

In an earlier study by Thu et al. [39] in the rural areas of Can Tho province, one positive pool of JEV was found from 22 048 mosquitoes, yielding a MIR of 0.05/1000 mosquitoes, similar to a MIR of 0.046/1000 *Cx. tritaeniorhynchus* in suburban Bangkok found by Gingrich et al. [40]. In the present study the MLE was 1.0/1000 mosquitoes and 1.6/1000 *Cx. tritaeniorhynchus*. Although it is possible that the higher MLE here is explained by higher infection rates in the mosquitoes in Can Tho city, it could also partially be due to higher sensitivity of the nested RT-PCR used. Also, all positive mosquito pools contained blood-filled mosquitoes as well as unfed. Thus, since it is possible that not all mosquitoes with JEV were actually infected, the actual infection rates may be lower than those calculated here. However, since the mosquitoes were all competent vectors for JEV, even presence of JEV in the blood meal indicates a risk of transmission.

Three of the PCR positive mosquito pools contained *Cx. tritaeniorhynchus*, a known competent vector for JEV [3]. The number of *Cx. tritaeniorhynchus* has been found to be significantly associated with the number of pigs in a household [10] and in the present study the positive pools were also consistently collected close to the pigs. One JEV positive mosquito pool contained *Cx. quinquefasciatus*, another competent vector. This species is anthropophilic and feed to a higher extent on humans than on pigs [41]. The positive mosquito pool was collected close to pigs, indicating that *Cx. quinquefasciatus* may have an important role as a bridge vector within Can Tho city.

The procedures used for mosquito handling and the established nested RT-PCR may provide a robust, economic and sensitive method for screening mosquito pools for JEV, which makes it suitable in tropical and low-income countries where the disease burden from mosquito-borne infections is high. TRIzol Reagent was used since it inactivates virus and helps preserving the RNA of RNA viruses for future analyses, even in samples stored at room temperature [42] and extraction with TRIzol Reagent does not require expensive equipment. We demonstrate that inhibition of PCR may cause problems for detection of arbovirus when screening mosquito samples, and that this should be taken into account. We also conclude that it is possible to avoid inhibition caused by mosquitoes simply by diluting samples.

The IgG sero-prevalence in the present study approached 100%. This was higher than the results by Lindahl et al. [43] when a commercial JEV ELISA kit was used for detection of JEV in sows in the rural area surrounding Can Tho city. However, ten years have passed between the samplings, which may explain the different results, and in addition, two different ELISA methods were used. The competitive IgG ELISA used in the present study has been shown to be cross-reactive with other flaviviruses in the JEV serological group, such as Murray Valley encephalitis virus and Kunjin virus [31], [32], but none of these flaviviruses have been demonstrated in southern Vietnam. Apart from JEV, dengue virus is the only vector-borne flavivirus in the region that infects mammals, and serological cross-reactions with JEV have been demonstrated [44]. However, cross-reactions with dengue virus occur to a lesser extent than with viruses in the JEV serological group [45], [46] and we therefore consider it unlikely that the positive results in our study would be due to cross reactions. Another flavivirus which could cause cross-reactions with JEV is Tembusu virus, which is present in Southeast Asia [47], although there are no reports of the virus in the Mekong delta region in Vietnam. More than half of the female pigs in the urban households were born at the farm where they were sampled and must thus have been infected at their present location. The negative and inconclusive results using the IgM ELISA could indicate that the JEV infections are not recent, or that infection with JEV occurred while the pigs were still partly protected by maternal IgG antibodies, thus inducing less IgM production [48]. JEV seropositivity has mainly been studied in humans in urban areas [35], [12] but since humans tend be mobile, pigs born in the city may be better indicators for JEV transmission within an urban environment. Notably, half of the female pigs had experienced reproductive symptoms that could be related to JEV infection. However, there are many other pathogens circulating in the Mekong Delta [49], [50] that can cause similar reproductive symptoms, although a previous study in Can Tho province could find an association between seropositivity to JEV and reduced reproductive performance in female pigs less than 1.5 years [44].

Whether humans are infected in the urban area or not, is not known. In the entire Can Tho city province the reported incidence of acute encephalitis has been on average 2.4 cases per 100 000 inhabitants during 2009–2012, with the majority of cases being in children under 6 months of age [51]. As in other endemic areas the number of clinical cases in adults is relatively low, due to the acquired natural immunity in the adult population [52], but the risk for clinical disease may be much higher for non-immune visitors from non-endemic areas. Vaccination against JEV is increasing in Vietnam although not all children are covered yet [14]. With increasing indications of risks for urban transmission of JEV there may be cause to revise vaccination policies.

In conclusion, the present study demonstrates the presence of JEV within an urban area by finding both serological evidence of widespread infections in pigs and mosquitoes PCR-positive for the virus.
